# Development and initial cohort validation of the Arthritis Research UK Musculoskeletal Health Questionnaire (MSK-HQ) for use across musculoskeletal care pathways

**DOI:** 10.1136/bmjopen-2016-012331

**Published:** 2016-08-05

**Authors:** Jonathan C Hill, Sujin Kang, Elena Benedetto, Helen Myers, Steven Blackburn, Stephanie Smith, Kate M Dunn, Elaine Hay, Jonathan Rees, David Beard, Sion Glyn-Jones, Karen Barker, Benjamin Ellis, Ray Fitzpatrick, Andrew Price

**Affiliations:** 1 Arthritis Research UK Primary Care Centre, Keele University, Keele, Staffordshire, UK; 2 Nuffield Department of Orthopaedics, Rheumatology & Musculoskeletal Sciences, Botnar Research Centre, University of Oxford, Oxford, Oxfordshire, UK; 3 Arthritis Research UK, London, UK; 4 Nuffield Department of Population Health, University of Oxford, Oxford, Oxfordshire, UK

**Keywords:** Outcome Assessment, Questionnaires, Validation studies, Cohort studies

## Abstract

**Objectives:**

Current musculoskeletal outcome tools are fragmented across different healthcare settings and conditions. Our objectives were to develop and validate a single musculoskeletal outcome measure for use throughout the pathway and patients with different musculoskeletal conditions: the Arthritis Research UK Musculoskeletal Health Questionnaire (MSK-HQ).

**Setting:**

A consensus workshop with stakeholders from across the musculoskeletal community, workshops and individual interviews with a broad mix of musculoskeletal patients identified and prioritised outcomes for MSK-HQ inclusion. Initial psychometric validation was conducted in four cohorts from community physiotherapy, and secondary care orthopaedic hip, knee and shoulder clinics.

**Participants:**

Stakeholders (n=29) included primary care, physiotherapy, orthopaedic and rheumatology patients (n=8); general practitioners, physiotherapists, orthopaedists, rheumatologists and pain specialists (n=7), patient and professional national body representatives (n=10), and researchers (n=4). The four validation cohorts included 570 participants (n=210 physiotherapy, n=150 hip, n=150 knee, n=60 shoulder patients).

**Outcome measures:**

Outcomes included the MSK-HQ's acceptability, feasibility, comprehension, readability and responder burden. The validation cohort outcomes were the MSK-HQ's completion rate, test–retest reliability and convergent validity with reference standards (EQ-5D-5L, Oxford Hip, Knee, Shoulder Scores, and the Keele MSK-PROM).

**Results:**

Musculoskeletal domains prioritised were pain severity, physical function, work interference, social interference, sleep, fatigue, emotional health, physical activity, independence, understanding, confidence to self-manage and overall impact. Patients reported MSK-HQ items to be ‘highly relevant’ and ‘easy to understand’. Completion rates were high (94.2%), with scores normally distributed, and no floor/ceiling effects. Test–retest reliability was excellent, and convergent validity was strong (correlations 0.81–0.88).

**Conclusions:**

A new musculoskeletal outcome measure has been developed through a coproduction process with patients to capture prioritised outcomes for use throughout the pathway and with different musculoskeletal conditions. Four validation cohorts found that the MSK-HQ had high completion rates, excellent test–retest reliability and strong convergent validity with reference standards. Further validation studies are ongoing, including a cohort with rheumatoid/inflammatory arthritis.

**Strengths and limitations of this study**

- A new musculoskeletal health questionnaire (MSK-HQ) has been successfully developed through a coproduction process with patients.
- The MSK-HQ captures key outcomes that were shown to be highly relevant to patients across a range of musculoskeletal conditions and settings.
- Promising measurement properties were found in four different musculoskeletal cohorts, with high completion rates, excellent test–retest reliability, and strong convergent validity with reference standards.
- Limitations of the study were the lack of a rheumatoid/inflammatory arthritis validation cohort and that the MSK-HQ's responsiveness has yet to be tested.

## Introduction

Taken together, osteoarthritis, inflammatory disorders and common musculoskeletal conditions such as back, neck, shoulder, hip and knee pains now represent the single greatest cause of years lived with disability.^1^ Finding ways to prevent this impact on quality of life from increasing is a significant and important challenge.^2^ In the UK, these conditions are primarily managed in primary care, with referral to interface clinics and secondary care for more complex management or specialist treatment and surgery such as rheumatology or joint replacement. Until recently, many musculoskeletal services have been provided within distinct, discrete silos of care that have failed to address the long-term nature of these conditions or the fact that many patients have multiple musculoskeletal symptoms in more than one region of the body.^3–5^ Evidence exists for a wide variation in service performance, with a lack of consistency and continuity of care across the clinical pathway and poor adherence to the National Institute of Health and Care Excellence's (NICE) Quality Standards of Care for musculoskeletal conditions.^6^ ^7^ Current outcome tools and data collection systems are disparate and fragmented across different healthcare settings, and as a consequence, although many healthcare commissioners are aiming to reorientate services from their traditional focus on acute and episodic care towards better prevention, self-care and integrated primary care,^8^ there is a lack of clinical tools that link together different parts of the clinical pathway.

Patient-reported outcome measures (PROMs), which are short, self-completed questionnaires designed to capture patient views about their health status,^9^ are ideally suited to areas such as musculoskeletal health where disease impact is not easily captured using biomarkers. PROMs are therefore increasingly valued for their use in evaluating the performance of musculoskeletal services alongside measures of patient safety, patient experiences and service indicators. One example of the ability of PROM data to act as a catalyst for raising standards has been evidenced through the UK's National PROMs Programme which provides online reports^10^ identifying the worst and best healthcare providers for four high-cost surgical procedures (hip and knee replacement, varicose vein removal and hernia repair). Building on early successes from this initiative, there have been growing calls for new and practical musculoskeletal PROMs that can measure musculoskeletal health status across the pathway and across different pain problems. The vision is for the routine and systematic use of a single musculoskeletal PROM throughout different parts of the service to drive forward quality improvement and ensure that exemplar services are identified and emulated.

The overall aim of this project was to develop and validate a new musculoskeletal PROM: the Arthritis Research UK Musculoskeletal Health Questionnaire (MSK-HQ). Prerequisites for this MSK-HQ were as follows: it should be coproduced with patients and clinicians to identify aspects of health that were meaningful to both; it should aim to provide a holistic view of the impact on a person's musculoskeletal health throughout the clinical pathway, and be applicable for use by different MSK health professionals; it should be generic across different MSK conditions and help identify individual treatment targets; it should be sensitive to change to enable longitudinal measurement and the monitoring of changes over time; it should demonstrate robust psychometric properties; and finally, it should be easily interpretable and feasible for use in routine, busy clinical practice.

In this study, we address the following three objectives: (1) identifying and prioritising key outcomes to include in the MSK-HQ; (2) developing the draft MSK-HQ through a process of face and content validity testing; and (3) the initial validation study to report the MSK-HQ's scoring, completion rate, test–retest reliability, convergent validity and internal consistency in primary and secondary care musculoskeletal cohorts.

## Methods

### Objective 1: identifying and prioritising key outcomes to include in the MSK-HQ

#### Scoping exercise

A brief scoping exercise was conducted by an experienced systematic reviewer to identify health outcome domains highlighted within primary and secondary research used to describe disease impact and characterise improvement for patients with arthritis, inflammatory conditions and musculoskeletal pain. Intervention studies were searched on the MEDLINE database from 1 January 2000 to 1 December 2013, and data were extracted using the following headings: author, date, clinical setting, domains used to characterise patients and the primary outcome. The purpose of this exercise was to identify a list of potentially relevant outcomes to inform the following consensus process.

### Consensus workshop

A consensus workshop with stakeholders from the UK musculoskeletal community was held to identify and prioritise key musculoskeletal outcome domains for inclusion in the MSK-HQ. Stakeholders (n=29) in attendance included patients (n=8; from primary care, orthopaedic and rheumatology services), clinicians (n=7; including general practitioners (GPs), physiotherapists, orthopaedists, rheumatologists and pain specialists), national musculoskeletal patient and professional body representatives (n=10) and musculoskeletal researchers (n=4). All participants provided informed written consent, and patient representatives were remunerated in line with INVOLVE guidance.^11^ The workshop used a nominal group technique^12^ with patients having an equal voice. Initially, a study presentation was given including information on the outcome domains identified from the literature review. Then small group discussions (including a dedicated patient group) were held to identify potential domains for inclusion, followed by a full group discussion, and blind vote to retain domains with broad consensus (defined as >50% of participants). Finally, individual participants ranked a final list of domains. Participants were also asked to discuss the maximum number of items within the MSK-HQ and the type of response options it would include.

### Objective 2: developing the draft MSK-HQ through a process of face and content validity testing

#### Face and content validity testing

Having obtained a final list of prioritised musculoskeletal outcome domains, single items for each domain were formulated using relevant existing outcome domain questionnaires, expertise within the team and an iterative process with patients to optimise the wording of items and to ensure that each question appropriately captured its respective prioritised domain (content validity). The formal iterative process to improve the MSK-HQ's face validity and content validity involved holding four focus groups, with six individual patients. The first two focus groups were held at Keele University with three patients, two of whom had osteoarthritis and the third had back pain. The next two focus groups were held at Oxford University with three patients, one with rheumatoid arthritis, and two with experience of orthopaedic surgery (hip and knee). In addition, before and after each workshop, the MSK-HQ was iteratively improved through a cognitive interview with each of the six patients using a combination of verbal probing and think-aloud methods^13^ to establish the tool's acceptability, feasibility, comprehension, readability and perceived responder burden.

#### Stakeholder acceptability

To determine the MSK-HQ's acceptability to the wider musculoskeletal community, a second workshop with the same stakeholders involved in the first consensus workshop was held to present the final candidate MSK-HQ prior to psychometric testing. A blind vote was used to confirm whether the stakeholders agreed that the measure was acceptable for validation testing (>80% agreement required) and to agree the context in which the MSK-HQ should and should not be used. The culmination of this process was a candidate MSK-HQ ready for psychometric testing.

### Objective 3: initial measurement properties of the MSK-HQ

#### Design and setting

##### Community physiotherapy cohort

A cross-sectional validation cohort was derived from consecutive consulters in community musculoskeletal physiotherapy clinics in five UK West Midlands towns (Middlewich, Congleton, Wombourne, Cheadle and Wolverhampton). These clinics provide individual, face-to-face treatments within the English National Health Service (NHS) for patients referred from their GP. Participants received usual physiotherapy care according to clinical need. Consecutive adult (≥18 years) consulters with a musculoskeletal disorder were invited to participate having received a study information pack with their community physiotherapy appointment. No further inclusion/exclusion criteria were used except that patients had to be referred to the clinic by their GP, with the expectation that the cohort would comprise patients with a heterogeneous range of diagnostic groups and unspecified presenting musculoskeletal problems. Participants completed the MSK-HQ and other measures before the start of treatment at the first clinic visit and again at the second visit (typically 2 weeks later) to investigate test–retest reliability of the tool.

##### Secondary care orthopaedic cohorts

Three validation cohorts were recruited from the Nuffield Orthopaedic Centre in Oxford by introducing the MSK-HQ into routine questionnaires used in the assessment pathway for patients listed for orthopaedic surgery for the knee, hip and shoulder. Adult participants (≥18 years) completed a standard set of questionnaires at their preoperative assessment clinic, and a subset completed the MSK-HQ ∼5 days later at home for MSK-HQ reliability testing.

###### Population descriptors

Baseline population descriptors were measured consistently across cohorts and included measures of demographic data (age, gender, work status) and pain characteristics: pain-related days off work over past 3 months, pain episode duration, number of pain-related visits to their GP in the past 3 months and outcome expectations (using a numerical response scale from 0 ‘it will get worse’ to 10 ‘it will be cured’).

###### Reference standard measures of construct validity

All patients completed questionnaires containing the candidate MSK-HQ and the EQ-5D-5L.^14^ The EQ-5D-5L utility score was calculated using the UK Crosswalk value set.^15^ In addition, the orthopaedic cohort patients completed the Oxford Hip Score (OHS), Oxford Knee Score (OKS), Oxford Knee Score-Activity & Participation Questionnaire (OKS-APQ) and Oxford Shoulder Score (OSS) for, respectively, hip, knee and shoulder problems, and the physiotherapy cohort completed the six-item Keele MSK-PROM.^16^

#### Test–retest reliability

To identify patients with stable symptoms, when patients completed the second MSK-HQ for test–retest reliability assessment, they also completed a patient global rating of improvement question, a recommended core outcome in chronic musculoskeletal and osteoarthritis trials.^17^ The item asked, “Overall compared to the start of treatment, my symptoms are: much better, better, same, worse, or much worse.” Stable patients were defined as those who reported that their symptoms were the ‘same’ at retest.

#### Scoring the MSK-HQ

To ensure simplicity of the MSK-HQ scoring, which stakeholders emphasised was important during the consensus workshops, scores from all 14 items are summed together (responses coded from ‘not at all’=4 to ‘extremely’=0, except for items 12 and 13, which have the response options in the reverse order) providing a range from 0 to 56, with higher scores indicating better MSK health status.

#### Statistical analysis

MSK-HQ acceptability was assessed using response rates and completeness of data by examining the normal distribution of MSK-HQ scores and floor and ceiling effects (<10% threshold). Complete case analyses were performed throughout the analyses for the MSK scores, with no imputation for missing values. A person–item map for a partial credit Rasch model was performed in order to build a hypothetical unidimensional line along which items and persons are located according to their difficulty and ability.^18^

The MSK-HQ items were tested for internal consistency using Cronbach's α to establish whether items may be treated as a single additive scale using baseline and retest data. The SEM was calculated using 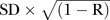.^19^

To examine test–retest reliability between MSK-HQ scores at baseline and retest, Kendall's coefficient of concordance (W) was calculated to examine individual item agreement,^20^ and the intraclass correlation coefficient (ICC—based on a two-way random effect, absolute agreement model) was used to test overall score agreement in the combined data set and as a sensitivity analysis for each individual cohort. An ICC above 0.70 is considered acceptable/good.^21^

To examine the convergent validity of the MSK-HQ against reference standard measures, we used Pearson's and Spearman's correlations between sum scores at baseline.^21^ The a priori hypothesis was that the MSK-HQ total score (higher=better) would follow a similar response pattern to those on reference standard scales.

The sample size for each validation cohort was calculated from the minimum number of patients recommended to investigate MSK-HQ test–retest reliability among a conservatively estimated 30% reporting stable symptoms. Using the Donner and Eliasziw^22^ approach for estimating sample size for reliability testing, we calculated that 102 people were needed for the physiotherapy cohort and orthopaedic cohorts combined, to detect a minimum acceptable ICC of 0.70, assuming a true ICC of 0.80, with a power of 80% and 5% significance level.

All analyses were conducted in STATA/IC V.14 (StataCorp LP., 2015), SPSS V.22 (IBM Corp, 2013) and Statistical software-R V.3.2.2 (The R Foundation for Statistics, 2015).

## Results

### Objective 1: identifying and prioritising key outcomes to include in the MSK-HQ

#### Scoping review and consensus workshop

The brief scoping review produced a list of over 75 existing outcome domains (available from the authors on request) from the literature. This was presented at the consensus workshop. Following the consensus process, participants identified and prioritised the following key outcomes for inclusion in the MSK-HQ (in priority order): severity of pain/stiffness (in the day and night), physical function (walking and dressing), physical activity level, pain interference (with work/daily routine and with social activities/hobbies), difficulty with sleep, fatigue/low energy levels, emotional well-being (anxiety and mood), understanding of diagnosis and treatment, confidence to self-manage (pain self-efficacy), independence and overall impact from symptoms. There were no marked differences in domain preferences between patients, clinicians and other stakeholders, and at the conclusion of the process, there was strong endorsement across the stakeholder community for the key domains that emerged. It was agreed that the MSK-HQ should include no more than 15 items and would use a response scale based on Likert ‘severity’ response options.

### Objective 2: developing the draft MSK-HQ through a process of face and content validity testing

A summary of the patients' feedback about the face validity, content validity, recall period, response scale, format and layout, sensitivity to change and application of the MSK-HQ is provided in table 1.

**Table 1:** Summary of patient feedback

General qualities	Patient feedback
Face validity	Patients felt most questions were relevant, easy to understand and answer. Patients tended to interpret the questions correctly as they were intended. Some queried if the MSK-HQ may be difficult for individuals with multiple MSK conditions, eg, ‘which condition do I talk about?’
Content validity	Patients considered that all items were highly relevant and important to their daily lives. Patients agreed that the MSK-HQ covered most prioritised domains they wanted. Other domains suggested included: Severity of and/or length of time with stiffness during the day and at night, Effectiveness of pain relief treatments/therapies, Impact on social activities, A general change in health question.
Recall period	Patients correctly used a 2-week recall period for most questions.
Response scale	All patients generally agreed that the response scale and descriptive responses were appropriate. The use of ‘extremely’ as the final response option was changed as it was not always appropriate.
Format and layout	Patients considered the layout and format to be appropriate. Some minor issues included: The MSK-HQ instructions and spacing of items, Response options descriptors should be close to tick boxes, Labelling of the items was improved. Patients did not generally notice the scoring codes for each item response. A few mentioned that they are used to see these on questionnaires and did not think that it was a problem having them included.
Sensitivity to change	Patients suggested that all domains were likely to change over time, depending on stage or severity of their condition: Domains most likely to change: walking, pain, sleep, physical activities and impact, Domains least likely to change: dressing and help needed.
Application and administration	Patients thought that the MSK-HQ would be useful to monitor health regularly. The generic nature of the questionnaire was mostly perceived to be a positive thing, so it can be used across different MSK conditions. Patients suggested that they would be happy to complete it themselves at home. Completion every 3 months was suggested as a suitable follow-up period.

On average, the MSK-HQ took around 2 min to complete. The MSK-HQ Flesch reading ease test score is 65.9, meaning that it is easily understood by 13–15-year-old students and is easier to read than many PROMs such as the EQ-5D-5L which scores 61.3.

The MSK-HQ is available online via General Info: http://isis-innovation.com/health-outcomes/ and a Licence request: http://process.isis-innovation.com/

**Figure 1:**
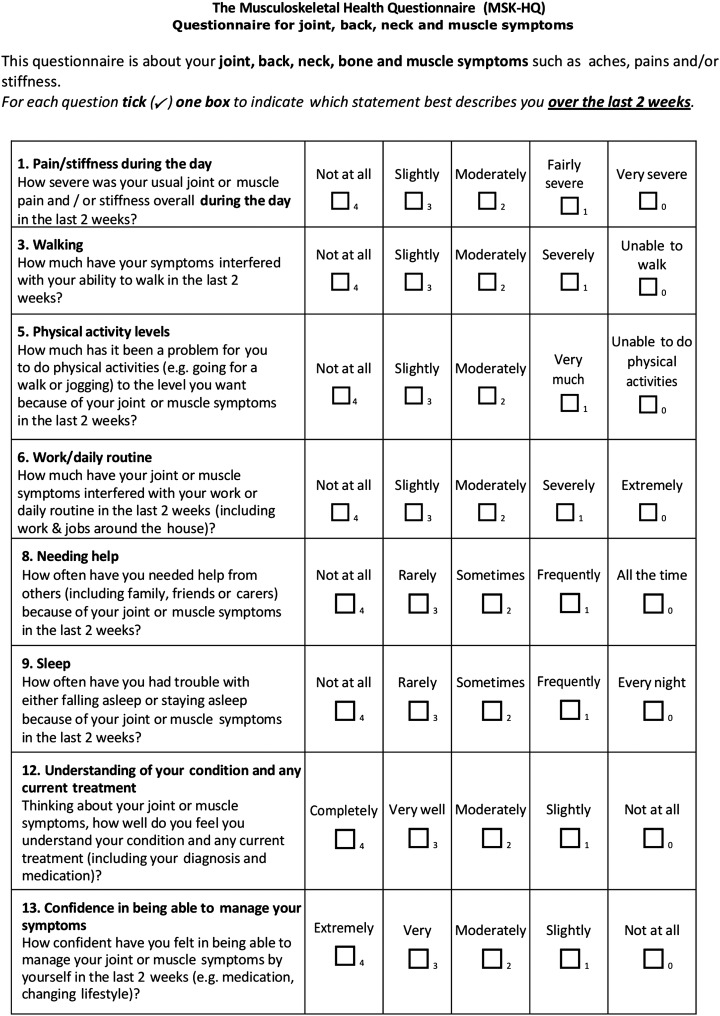
Example items from the MSK-HQ. MSK-HQ, Musculoskeletal Health Questionnaire.

Examples of the MSK-HQ items are shown in figure 1.

### Objective 3: initial measurement properties of the MSK-HQ

#### Study sample

There were 570 patients in total who consented to participate in the 4 studies (210 physiotherapy patients, 150 hip, 150 knee, and 60 shoulder). Baseline population characteristics for the overall sample and for each cohort are summarised in table 2, showing a mean age of 56.99 years (SD 16.54) with 65.19% women. The median pain episode duration was 6.58 months (SD 4.42), and the mean EQ-5D-5L utility score was 0.49 (SD 0.26).

**Table 2:** Baseline characteristics overall and by each cohort (values represent mean (SD) unless otherwise indicated)

Variable	All participants, n=570	Physio, n=210	Hip, n=150	Knee, n=150	Shoulder, n=60
Demographic variables					
Age (years)	56.99 (16.54)	53.53 (15.45)	55.62 (17.21)	65.68 (13.80)	51.54 (17.15)
Sex, n (%) female	313 (65.19)	112 (53.59)	88 (60.69)	89 (62.24)	24 (40.00)
Employment status, n (%) yes working	263 (48.88)	126 (60.29)	64 (45.07)	40 (30.53)	33 (58.93)
Taken time off work for pain, n (%)	56 (21.29)	27 (21.42)	15 (23.43)	8 (20.00)	6 (18.18)
Pain duration (months)	6.58 (4.42)	4.84 (2.95)	8.41 (5.76)	8.16 (4.69)	9.13 (4.13)
No. of pain-related visits to GP, past 3 months	1.39 (1.45)	1.53 (0.93)	1.47 (2.05)	1.37 (1.45)	0.73 (0.99)
Outcome expectations (NRS)	9.25 (1.63)	8.38 (1.77)	9.82 (1.18)	9.79 (1.27)	9.67 (1.56)
Clinical variables					
MSK-HQ total score	28.62 (9.61)	30.54 (9.56)	24.93 (8.27)	27.54 (9.03)	33.48 (10.54)
EQ-5D-5L utility score	0.49 (0.26)	0.55 (0.25)	0.40 (0.24)	0.45 (0.26)	0.56 (0.25)
Keele MSK-PROM		17.44 (4.45)			
OHS			20.4 (8.62)		
OKS				20.89 (8.84)	
OSS					29.62 (10.34)

Missing data for MSK-HQ score: all participants, n=33 (5.8%); Physio cohort, n=5 (2.4%); Hip cohort, n=4 (2.7%); Knee cohort, n=22 (14.7%); Shoulder cohort, n=2 (3.3%).

EQ-5D-5L, EuroQol 5 level; MSK-HQ, Musculoskeletal Health Questionnaire; NRS, numerical rating scale; OHS, Oxford Hip Score; OKS, Oxford Knee Score; OSS, Oxford Shoulder Score.

#### MSK-HQ acceptability/completion rates

**Figure 2:**
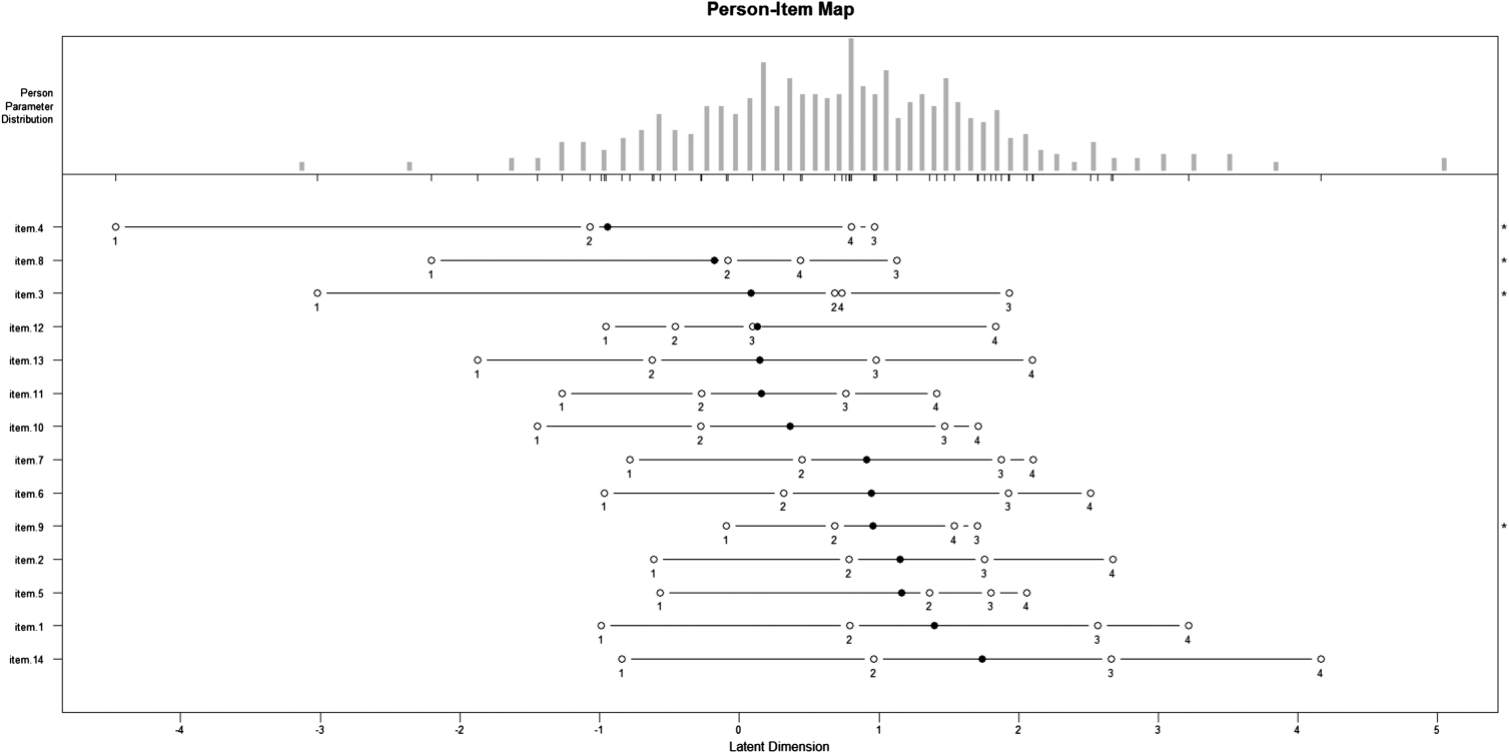
A person–item map for the Rasch partial credit model presents item response difficulty.

The MSK-HQ was acceptable to patients, with complete MSK-HQ data available for 537/570 patients (94.2%). Across the Hip, Physiotherapy, and Shoulder cohorts, there was around 3% missing data (see table 2), but the proportion of missing data was substantially higher in the Knee cohort at 14.7%, as data entry was not checked in clinic. In data for the four cohorts combined, the best completed MSK-HQ item was the ‘walking’ (item 3) with 4/570 (0.07%) missing responses, while the ‘fatigue/low energy’ (item 10) had the most missing responses 9/570 (1.6%). Within the knee cohort (n=150), missing responses were higher than for other cohorts but were spread fairly evenly across all 14 MSK-HQ items varying from 3/150 people (2%) for the ‘walking’, ‘social activities’ and ‘sleep’ items to 7/150 people (4.7%) for the ‘understanding of condition’ item. The person–item map for a partial credit Rasch model revealed that across the combined cohorts, the most difficult item to get a lower severity score was ‘overall impact’ (item 14) and that ‘washing/dressing’ (item 4) was the easiest item to get a lower severity score (see figure 2). No weighting was given to any items in order to ensure that the MSK-HQ is simple to use and interpret in clinical practice. The MSK-HQ scores for all the cohorts combined were normally distributed with an overall mean score of 28.62 (9.61) from a possible range of 0–56. No floor or ceiling effects were observed. Within the four cohorts, the Hip cohort had the worst overall MSK health status with a mean (SD) MSK-HQ total score of 24.93 (8.27). Overall MSK health status was about 3 points more favourable across each of the other three cohorts with mean (SD) MSK-HQ scores for Knee, Physiotherapy and Shoulder cohorts being 27.54 (9.03), 30.54 (9.56) and 33.48 (10.54), respectively. The SEM for the MSK-HQ was 5.52.

#### Internal consistency

Analysis of internal consistency demonstrated that the total score can be adequately considered as one scale, with a mean Cronbach's α at a baseline of 0.88. α Values for each individual item were similar and are provided in table 3. The item on ‘interference with work/daily routine’ was the most correlated item to the total MSK-HQ score (0.76), and responses for two items (understanding your condition and confidence to self-manage) were shown to correlate weakly (−0.04 and 0.32, respectively) with the total MSK-HQ score. The retest data showed similar patterns of results.

**Table 3:** Internal consistency of the MSK-HQ at baseline and retest

	Baseline, n=537			Retest, n=376			Baseline and retest	
Item	Mean (SD)	r_item-rest_	α=	Mean (SD)	r_item-rest_	α=	N	Kendall's W
MSK-HQ Total (0, 56)			0.88			0.92	358	0.91
1. Pain/stiffness during the day	1.52 (0.91)	0.69	0.87	1.71 (0.89)	0.76	0.91	379	0.83
2. Pain/stiffness at night	1.65 (1.09)	0.60	0.87	1.96 (1.12)	0.65	0.92	379	0.87
3. Walking	2.11 (1.16)	0.57	0.88	2.23 (1.18)	0.69	0.91	380	0.89
4. Washing/dressing	2.77 (1.02)	0.60	0.87	2.89 (0.97)	0.64	0.92	382	0.86
5. Physical activity levels	1.52 (1.14)	0.56	0.88	1.72 (1.29)	0.70	0.91	379	0.83
6. Work/daily routine	1.81 (1.03)	0.76	0.87	2.11 (1.04)	0.81	0.91	381	0.83
7. Social activities and hobbies	1.80 (1.11)	0.63	0.87	2.09 (1.17)	0.74	0.91	381	0.82
8. Needing help	2.62 (1.21)	0.65	0.87	2.70 (1.19)	0.73	0.91	380	0.88
9. Sleep	1.73 (1.28)	0.56	0.88	1.97 (1.35)	0.60	0.92	382	0.90
10. Fatigue or low energy	2.22 (1.08)	0.63	0.87	2.27 (1.07)	0.71	0.91	381	0.87
11. Emotional well-being	2.47 (1.16)	0.64	0.87	2.65 (1.12)	0.70	0.91	382	0.84
12. Understanding condition	2.60 (1.10)	−0.04	0.90	2.95 (0.80)	0.10	0.93	379	0.72
13. Confidence in managing	2.39 (0.99)	0.32	0.89	2.50 (0.94)	0.41	0.92	382	0.72
14. Overall impact	1.42 (0.88)	0.74	0.87	1.59 (0.96)	0.79	0.91	381	0.82

n is the number of individuals with complete scales.

r_item-rest_ is the correlation between an item and the scale that is formed by all other items.

α=Cronbach's α of the scale excluding all but one of the items, except where ‘Total’ indicates Cronbach's α for complete scale.

#### Test–retest reliability

There were 370/537 patients (70.0%) with retest MSK-HQ data available with a mean (SD) time interval of 5.92 (4.63) days. There were 245 (66.2%) patients reporting ‘stable’ symptoms between the two time points, with 73 (19.8%) reporting being ‘better’ and 52 (14%) ‘worse’. Within the group with ‘stable’ symptoms, the MSK-HQ total score agreement ICC was 0.84 (95% CI 0.77 to 0.89, n=226), demonstrating ‘excellent’ reliability. The sensitivity analysis for each individual cohort revealed that the ICC within the Hip cohort was 0.91 (95% CI 0.85 to 0.95, n=60), Knee cohort was 0.79 (95% CI 0.67 to 0.87, n=63), Physiotherapy cohort was 0.80 (95% CI 0.45 to 0.91, n=79) and Shoulder cohort was 0.93 (95% CI 0.84 to 0.97, n=24). Kendall's coefficient of concordance for individual item agreement in the combined data set ranged from 0.72 for the ‘understanding of condition’ and ‘confidence in managing symptoms’ items to 0.90 for the ‘sleep’ item. Details of inter-rater agreement for each of the 14 items are given in table 3.

#### Convergent validity

Pearson's and Spearman's rank correlations of the MSK-HQ with the EQ-5D-5L for the overall combined data were strong, being 0.80 and 0.81, respectively. Table 4 demonstrates strong correlations between the MSK-HQ and reference standards for each of the four cohorts, including the MSK-PROM, OHS, OKS and OSS, in particular with the OKS and OSS with Spearman's of 0.88 and 0.86, respectively.

**Table 4:** Convergent construct validity—correlations between reference standards

	Comparator	Index	Baseline		
			N	Spearman's correlation	Pearson's correlation
				s=(95% CIs)	r=(95% CIs)
MSK total (0, 56)	OHS (0, 48)	Hip	130	0.83 (0.77 to 0.88)	0.83 (0.77 to 0.88)
	OKS (0, 48)	Knee	125	0.88 (0.83 to 0.91)	0.89 (0.84 to 0.92)
	OSS (0, 48)	Shoulder	53	0.86 (0.78 to 0.92)	0.87 (0.79 to 0.93)
MSK total (0, 56)	EQ-5D-5L Index (−0.59, 1)	Total	525	0.81 (0.78 to 0.84)	0.80 (0.76 to 0.83)
		Hip	141	0.76 (0.68 to 0.82)	0.77 (0.69 to 0.83)
		Knee	123	0.78 (0.70 to 0.84)	0.75 (0.67 to 0.82)
		Shoulder	58	0.84 (0.74 to 0.90)	0.81 (0.70 to 0.89)
		Physio	203	0.82 (0.77 to 0.86)	0.81 (0.76 to 0.85)
MSK total (0, 56)	MSK-PROM (0, 30)	Total	203	0.81 (0.75 to 0.85)	0.82 (0.77 to 0.86)

## Discussion

This study describes the successful development and initial psychometric validation of the MSK-HQ. This new outcome measure has been coproduced with patients and clinicians to measure the holistic impact of an MSK condition on a person's health, regardless of the location of their MSK pain or where on the clinical pathway an individual is currently receiving care. The first phase of the project successfully identified and prioritised key outcomes that a broad range of MSK patients and clinicians ranked as the most important for identifying and monitoring the impact from an MSK condition on overall MSK health status. These domains included severity of pain/stiffness (in the day and at night), physical function (walking and dressing), physical activity level, symptom interference (with work/daily routine and with social activities/hobbies), difficulty with sleep, level of fatigue/low energy levels, emotional well-being (anxiety and mood), understanding of diagnosis and treatment, confidence to self-manage (pain self-efficacy), independence and overall impact from symptoms. The wording for single items to capture each of these domains was successfully optimised through a process of face and content validity testing with users, resulting in 14 items that patients with a range of MSK conditions felt were ‘highly relevant’ to their lives and ‘easy to understand’.

Our validation study included 570 MSK patients from 4 different cohorts with a range of MSK conditions from primary/community and secondary care settings. The results demonstrated that the MSK-HQ was well completed, has excellent test–retest reliability and has strong convergent validity with reference standards. The findings were consistent across the four cohorts suggesting promising initial cross-sectional psychometric properties of the MSK-HQ. As might be expected, patients' MSK health status (measured by the MSK-HQ total score) was shown to be worst among secondary care patients awaiting hip surgery (mean=24.93) and knee surgery (mean=27.54), and was less severe among those receiving community physiotherapy (mean=30.54). While the MSK-HQ is a multidimensional measure, its high internal consistency across items (Cronbach's α of 0.88) suggests that it can be considered as one scale for overall MSK health status with the MSK-HQ total score. To ensure simplicity of the MSK-HQ scoring in routine clinical practice, which emerged as important during the consensus workshops, scores from individual items are summed together, providing a range from 0 to 56. The MSK-HQ overall score is not a score of a single construct (reflective model), but a sum of items from different domains measuring overall musculoskeletal health status (formative model). Alternative scoring approaches including weighting items were discussed at the second stakeholder workshop, and it was agreed that first, a non-weighted approach was better suited to using the tool in routine practice, and second that the provision of a single additive scale was clinically useful in helping to evaluate the overall impact of the musculoskeletal condition on the individual. The study identified that the test–retest reliability of the MSK-HQ's total scores among ‘stable’ patients between the baseline and retest time points (using ICCs) was ‘excellent’ overall. In addition, as a sensitivity analysis, we examined the test–retest reliability separately for each of the 4 cohorts, which found that the ICC varied from 0.79 to 0.93. It should be noted, however, that it is unwise to use these figures to directly compare the reliability of the tool in the different cohorts due to the potential for bias as the study was not powered for this sensitivity analysis and the proportion of ‘stable’ patients differed across the four cohorts. Finally, the strong correlations with different single MSK condition reference standards, particularly with the Shoulder, Knee and Hip cohort reference standards (OSS=0.86, OKS=0.88 and OHS=0.83), show the potential for the MSK-HQ to capture overall MSK health status across different MSK conditions instead of relying on existing condition-specific measures.

In order for healthcare services and individuals with MSK conditions to better manage and monitor their own health, appropriate clinical tools are required that can capture the overall impact from fluctuating symptoms.^23^ Previous research has sought to identify key outcome domains for different musculoskeletal conditions but have not sought to have one list of outcome domains that can capture the overall impact for all MSK conditions. For example, a work in 1998 by Deyo *et al*^24^ recommended the following core outcome domains for low back pain disorders: pain (severity and frequency), back-related function, generic well-being, difficulty with social role/work and patient satisfaction with care. In 2014, four more domains were added to this list: pain interference, depression, sleep disturbance and catastrophising.^25^ For patients with osteoarthritis, recommended outcome domains include pain, functional impairment and patient's global assessment of change.^26–28^ Separately, the International Classification of Functioning, Disability and Health has made individual recommendations for different MSK conditions such as low back pain, chronic widespread pain, ankylosing spondylitis, osteoporosis, osteoarthritis and rheumatoid arthritis with the most common domains across conditions being symptom severity (pain intensity), function (physical function, social function, work function), generic well-being/quality of life, patient's global assessment of change, emotional functioning, independence and patient satisfaction.^29^ It can be seen that the domains included in the MSK-HQ are largely consistent with all of the above recommendations, although the MSK-HQ does not measure domains such as patient satisfaction or global assessment of change which are typically captured at a single post-treatment time point and not longitudinally over time.

A key vision of the MSK-HQ was to fill the current gap for a single broad health status measure instead of relying on generic health tools such as the EQ-5D-5L which have been shown to be less sensitive to change in MSK populations.^16^ One key requirement for the MSK-HQ yet to be tested is whether it is more sensitive to change than the EQ-5D-5L. Follow-up data are currently being collected, to be reported separately in due course. Such a tool would have strong potential in helping to overcome current challenges in driving forward MSK health service improvements caused by the use of so many different PROMs across the pathway, despite a common entry point for different MSK conditions. The use of the MSK-HQ as a standard summative PROM across the MSK pathway is initially supported by the results of this study, although further research to examine the responsiveness and applicability of this tool in other musculoskeletal patient populations is recommended.^30^

In many long-term conditions, such as diabetes or asthma, PROMs are also used to guide treatment. This too was part of the vision for the MSK-HQ, to capture an individual's MSK health status at any given time and thereby enable patients and their clinicians to monitor progress over time and response to treatment. Individual MSK-HQ items capturing ‘sleep’ or ‘physical activity’ could also enable specific patient needs to be tracked over time and support the reporting of key issues to clinical teams, thereby facilitating better shared decision-making in consultations. Further strengths of the MSK-HQ are its coproduction with patients, using domains which have high face validity, are easy to understand as well as being reliable and valid in heterogeneous MSK populations. However, a clear weakness of this study is the lack of a rheumatology or pain clinic validation cohort, although separate work is in progress to test the MSK-HQ within a rheumatology setting and data will be available soon. Another weakness is that missing item rules and minimal clinically important differences are not yet available for this measure, although future studies will seek to address these issues. It is interesting to note that MSK-HQ completion rates were below, or at, 3% when the tool was completed and checked in clinic but were nearly 15% in the knee cohort where patients completed the tool unsupervised at home. It will be important for future studies to test whether electronic data capture rather than the paper-based questionnaires used in this study is able to reduce the number of missing items in contexts where the tool is completed unsupervised.

Important next steps for this research are to examine the factor structure of the MSK-HQ as well as its responsiveness in comparison to condition-specific measures such as the Oxford Hip and Knee Scores and generic health status measures such as the EQ-5D-5L. Future research opportunities for the MSK-HQ include its potential to help in reviewing patients MSK health status in primary care chronic disease review clinics, and testing its usefulness as a consultation prompt and care planning tool to shape musculoskeletal consultation conversations and ensure that individual issues are addressed.

## Conclusion

A new PROM for a broad range of MSK conditions has been successfully developed, called the MSK-HQ. This novel PROM contains 14 items that capture key outcomes that patients with a range of MSK conditions have prioritised as important for use across the clinical pathway. The MSK-HQ has also undergone initial psychometric testing in four different MSK cohorts and demonstrated high completion rates, excellent test–retest reliability and strong convergent validity with reference standards, including the EQ-5D-5L, and Oxford Hip, Knee and Shoulder scores. Ongoing follow-up studies will examine the responsiveness and factor structure of the MSK-HQ in the future.
